# Association Between Prenatal Cannabis Exposure and Child Health Care Use: A Retrospective Cohort Study in Ontario, Canada

**DOI:** 10.1016/j.jpedcp.2025.200151

**Published:** 2025-06-02

**Authors:** Gabrielle Pratt Tremblay, Arum Han, Ewa Sucha, Helen Hsu, Jessy Donelle, Daniel J. Corsi

**Affiliations:** 1 School of Epidemiology and Public Health, Faculty of Medicine, University of Ottawa, Ottawa, ON, Canada; 2 Children's Hospital of Eastern Ontario Research Institute, Ottawa, ON, Canada; 3 Faculty of Medicine, University of Ottawa, Ottawa, ON, Canada; 4 ICES uOttawa, Ottawa, ON, Canada; 5 Kingston Health Sciences Centre, Kingston, ON, Canada

**Keywords:** cannabis, marijuana, pregnancy, health services, health administrative databases, retrospective, substance use, BORN

## Abstract

**Objective:**

Cannabis use among expectant mothers has increased steadily over the past two decades. We compared the long-term health services use of offspring prenatally exposed to cannabis to that of matched, unexposed offspring.

**Study design:**

We conducted a retrospective cohort study using linked perinatal and health administrative databases of all live, singleton births in Ontario, Canada hospitals between April 1, 2007 and March 31, 2012. Infants were followed until March 31, 2017, with a primary outcome of primary care visits up to age 10. Secondary outcomes included rates of outpatient psychiatrist visits, emergency department visits and hospitalizations. We used adjusted Poisson regression to assess differences in rates of health service use between children with and without exposure to prenatal cannabis.

**Results:**

We included 508 025 infants, 3248 (0.6%) had cannabis exposure. Prenatal cannabis use was associated with a decreased rate of primary care physician visits (adjusted rate ratio [aRR]: 0.86, 95% confidence interval [CI]: 0.84-0.87) and an increase in the rate of outpatient psychiatrist visits (aRR: 1.29, 95% CI: 1.00-1.66), emergency department visits (aRR: 1.05, 95% CI: 1.03-1.08), and hospitalizations (aRR: 1.12, 95% CI: 1.04-1.20). Among preterm offspring, cannabis was associated with a decrease in primary care visits but no difference in other visits. Among those in the highest income quintiles, cannabis use was associated with a two-fold increase in the rate of outpatient psychiatrist visits.

**Conclusions:**

Offspring exposed to prenatal cannabis receive fewer primary care visits but have increased rates of visiting health care specialists past the neonatal period.

Cannabis is one of the most widely used drugs in Canada, and its use has steadily increased across many subpopulations, including pregnant women.^1^^,^^2^ Among Canadians, between 2% and 5% of women self-report cannabis use in pregnancy.^3^ However, rates are substantially higher among women in certain age groups,^4^ and self-reports may underestimate use due to the stigma around disclosure in pregnancy.^5^ Available evidence suggests that pregnant individuals may use cannabis as a way to address health concerns, including pain, nausea, anxiety, depression, and other problems.^6^, ^7^, ^8^, ^9^ In our previous qualitative interviews with patients, we noted that individuals who regularly consume cannabis before conception may be more likely to continue their use, either out of habit or to maintain a sense of normalcy throughout their pregnancy.^10^

Cannabinoids can reach the fetal bloodstream via the placenta.^11^^,^^12^ These cannabinoids can disrupt the fetal endocannabinoid system, which is active from early stages of embryonic development and plays a critical role in neurodevelopment into adulthood. Due to its lipophilic nature, the principal psychoactive component of cannabis, delta-9 tetrahydrocannabinol, has a long half-life, which results in extended exposure to fetal tissues even after the mother stops consuming cannabis.^13^^,^^14^ Accumulating evidence suggests that cannabis use during pregnancy may be associated with adverse perinatal and neonatal outcomes, including stillbirth,^15^^,^^16^ preterm birth,^15^^,^^17^, ^18^, ^19^, ^20^ growth restrictions^17^^,^^21^, ^22^, ^23^, and increased admission to the neonatal intensive care unit.^17^^,^^18^^,^^20^^,^^24^ These outcomes are often associated with more extended hospital stays after delivery and more complex health and social needs throughout early childhood.^25^ However, little is known about the patterns of long-term use of health services of children prenatally exposed to cannabis in Canada. Using Ontario's population-based birth registry and linked health administrative databases, we aimed to compare the rates of long-term health services use among offspring associated with prenatal cannabis use vs no use.

## Methods

### Study Design

We conducted a population-based retrospective cohort study of all live, singleton births to Ontario, Canada residents, between April 1, 2007, and March 31, 2012. An analysis examining child neurodevelopmental outcomes in a cohort with follow-up from 18 months was previously published.^26^ This paper describes the use of health services among children with and without prenatal cannabis exposure using the entire birth cohort from birth to age 10. We report the study according to the REporting of studies Conducted using Observational Routinely collected Data reporting guidelines.^27^

### Data Sources

The study was conducted at Institute for Clinical Evaluative Sciences (ICES), an independent, nonprofit research institute whose legal status under Ontario's health information privacy law allows it to collect and analyze health care and demographic data without consent for health system evaluation and improvement. We used the Better Outcomes Registry & Network (BORN, www.bornontario.ca) Niday Perinatal Database to define the birth cohort. The BORN Niday Perinatal Database includes a historical subset of the BORN registry, covering perinatal data from hospital births in Ontario from 2006 to 2012. Routine data collected in BORN includes information on maternal demographics and health behaviors, pre-existing health problems, obstetric complications, and birth outcomes. Data were collected from medical records, clinical forms, and patient interviews. In addition to the cohort, prenatal cannabis exposure and covariates related to pregnancy and birth outcomes were ascertained from this database. The birth cohort was subsequently linked to health administrative databases using unique encoded identifiers and analyzed at ICES. The study was approved by the research ethics boards of the Ottawa Health Science Network and the Children's Hospital of Eastern Ontario.

### Study Population

The study cohort was derived by accessing multiple databases that record the provision of health care in Ontario (Supplemental Appendix). We included all hospital births from 20 weeks of gestation weighing at least 500g and occurring in the province between April 1, 2007 and March 31, 2012. Infants were followed through linked databases until March 31, 2017, death or migration out of province using the ICES Registered Persons Database. We excluded mothers older than 50, those ineligible for the Ontario Health Insurance Plan (OHIP) during pregnancy, individuals who did not reside in Ontario for 2 years before pregnancy or had multifetal pregnancies (Supplemental Table S1; available at www.jpeds.com). Infants were excluded if they were stillborn, did not have a valid health card number at birth or within 60 days, were born to non-Ontario residents, or due to missing gestational age or incomplete database linkage.

### Exposure

Maternal cannabis use in pregnancy included either therapeutic or recreational use. Cannabis use in the current pregnancy was reported to providers during 1st trimester visits, which usually occur by the 11th gestational week^26^ but could also have been identified from clinical histories at admission for labor and delivery. At the first prenatal visit, providers complete a standardized perinatal record, which includes information about cannabis and substance use in the current pregnancy. Data from the perinatal and labor and delivery records were abstracted into the BORN Niday Perinatal Database. Cannabis use reporting in the perinatal database was well validated against clinical records.^28^ We found that BORN had a sensitivity of 97% (95% confidence interval [CI]: 93%-99%) and a specificity of 94% (95% CI: 91%-96%) compared to clinical records, and the positive predictive value was 90% (95% CI: 85% - 94%).^18^

### Outcomes

The primary outcome was the number of primary care visits during the follow-up period, which was identified using the OHIP claims database. Primary care visits were defined as any encounter with a family physician or pediatrician based on provider codes (Supplemental Table S2; available at www.jpeds.com). The secondary outcomes included the number of outpatient psychiatrist visits, identified using provider codes in the OHIP database, emergency department (ED) visits and inpatient hospitalizations, determined from the NACRS and DAD, respectively during the follow-up period (Supplemental Appendix; available at www.jpeds.com). If an ED visit led to hospitalization, it was counted as one ED visit and one hospitalization to reflect the frequency of services used.

### Covariates

Covariates of interest included maternal age, education level, prenatal substance use (including alcohol, cocaine, hallucinogens, methadone, opioids, and prescribed medication), rurality, household income, parity, antenatal care provider status, smoking, diagnosis of mood/anxiety disorder, diabetes, hypertension, asthma, and heart conditions. Maternal age was categorized into: <20, 20-24, 25-29, 30-34 and 35 and older. Education was categorized into quartiles, with one representing the lowest and four representing the highest level. Prenatal consumption of alcohol, cocaine, hallucinogens, methadone, opioids, or prescribed medication (eg, oxycodone, codeine) were each dichotomized (yes/no) and restricted to any consumption during the gestational period. Household income was categorized into quantiles, with one representing the lowest and five representing the highest level. Parity was categorized into nulliparous, primiparous, or multiparous. Antenatal care provider status, maternal smoking status and pre-existing maternal conditions, including mood/anxiety disorder, diabetes, hypertension, asthma, and heart conditions (eg, coronary heart disease, arrythmia), were each dichotomized (yes/no). Mood/anxiety disorder was defined as any psychiatric diagnosis recorded in the last two years before the last menstrual period.

### Statistical Analyses

We conducted descriptive analyses to describe the differences in demographic and clinical characteristics between mothers who consumed cannabis during pregnancy and those who abstained (Table I). This analysis was performed again after applying coarsened exact matching (CEM). We used CEM to reduce bias and imbalance in covariates across cannabis use.^29^ To achieve a balanced dataset, cannabis users were 1:k matched to nonusers within categories of the covariates mentioned above. Weights were applied to equalize the number of cannabis-using and non-using subjects in each stratum. Standardized mean differences (SMDs) were used to assess the balance in the distribution of baseline covariates between exposed and unexposed subjects before and after matching.^30^ An absolute SMD above 0.10 was considered a meaningful imbalance in the covariate distribution between the groups.

**Table I:** Baseline Characteristics of the Original and Matched Cohorts, Separated by Exposure Status and Compared Using Standardized Differences

Characteristic (%)	Birth cohort by prenatal cannabis exposure (n = 508 025)			CEM cohort by prenatal cannabis exposure (n = 175 700)		
	Unexposed n = 504 777	Exposed n = 3248	Standardized mean difference	Unexposed n = 173 269	Exposed n = 2431	Standardized mean difference
Maternal age, mean (SD)	30 (27-34)	23 (19-27)	1.11	23.6 (5.4)	23.4 (5.5)	0.02
Maternal age (categories)						
<20	18 199 (3.6%)	843 (26.0%)	0.66	47 327 (27.3%)	664 (27.3%)	0.00
20-24	63 600 (12.6%)	1183 (36.4%)	0.58	65 288 (37.7%)	916 (37.7%)	0.00
25-29	138 307 (27.4%)	723 (22.3%)	0.12	36 421 (21.0%)	511 (21.0%)	0.00
30-34	173 555 (34.4%)	327 (10.1%)	0.61	16 108 (9.3%)	226 (9.3%)	0.00
35+	111 116 (22.0%)	172 (5.3%)	0.50	8125 (4.7%)	114 (4.7%)	0.00
Maternal education, mean (SD)∗	0.51 ± 0.13	0.43 ± 0.12	0.63	0.43 ± 0.12	0.43 ± 0.12	0.00
Maternal education (quartiles)∗						
1 (lowest)	4022 (0.8%)	73 (2.2%)	0.12	82 465 (47.6%)	1157 (47.6%)	0.00
2	119 353 (23.6%)	1475 (45.4%)	0.47	47 184 (27.2%)	662 (27.2%)	0.00
3	130 826 (25.9%)	895 (27.6%)	0.04	2780 (1.6%)	39 (1.6%)	0.00
4 (highest)	15 902 (3.2%)	69 (2.1%)	0.06	38 452 (22.2%)	536 (22.1%)	0.00
Missing	234 674 (46.5%)	736 (22.7%)	0.52	2388 (1.4%)	37 (1.5%)	0.01
Pre-existing maternal conditions						
Diabetes	10 715 (2.1%)	55 (1.7%)	0.03	1426 (0.8%)	20 (0.8%)	0.00
Hypertension	14 041 (2.8%)	29 (0.9%)	0.14	927 (0.5%)	13 (0.5%)	0.00
Asthma	23 197 (4.6%)	405 (12.5%)	0.28	18531 (10.7%)	260 (10.7%)	0.00
Heart disease	2722 (0.5%)	24 (0.7%)	0.03	642 (0.4%)	9 (0.4%)	0.00
Maternal psychiatric disorders						
Mood/anxiety disorders	136 537 (27.0%)	1449 (44.6%)	0.37	71 560 (41.3%)	1004 (41.3%)	0.00
Substance use disorder	7750 (1.5%)	604 (18.6%)	0.59	19 957 (11.5%)	280 (11.5%)	0.00
Maternal substance use history						
Alcohol	453 (0.1%)	129 (4.0%)	0.28	1354 (0.8%)	19 (0.8%)	0.00
Cocaine	674 (0.1%)	450 (13.9%)	0.56	5916 (3.4%)	83 (3.4%)	0.00
Hallucinogens	59 (0.0%)	78 (2.4%)	0.22	71 (0.0%)	<6†	0.00
Methadone	1089 (0.2%)	149 (4.6%)	0.29	2922 (1.7%)	41 (1.7%)	0.00
Opioids	481 (0.1%)	169 (5.2%)	0.32	1069 (0.6%)	15 (0.6%)	0.00
Prescription medication	14 953 (3.0%)	371 (11.4%)	0.33	12 259 (7.1%)	172 (7.1%)	0.00
Rurality						
Urban	454 714 (90.1%)	2667 (82.1%)	0.23	146 256 (84.4%)	2052 (84.4%)	0.00
Rural	50 026 (9.9%)	580 (17.9%)	0.23	27 013 (15.6%)	379 (15.6%)	0.00
Income quintile						
1 (lowest)	106 072 (21.0%)	1454 (44.8%)	0.52	80 826 (46.6%)	1134 (46.6%)	0.00
2	99 429 (19.7%)	741 (22.8%)	0.08	40 128 (23.2%)	563 (23.2%)	0.00
3	104 029 (20.6%)	425 (13.1%)	0.20	21 953 (12.7%)	308 (12.7%)	0.00
4	108 515 (21.5%)	342 (10.5%)	0.30	17 035 (9.8%)	239 (9.8%)	0.00
5 (highest)	84 267 (16.7%)	231 (7.1%)	0.30	11 404 (6.6%)	160 (6.6%)	0.00
Missing	2465 (0.5%)	55 (1.7%)	0.12	1924 (1.1%)	27 (1.1%)	0.00
Parity						
Nulliparous	214 290 (42.5%)	1856 (57.1%)	0.30	106 199 (61.3%)	1490 (61.3%)	0.00
Primiparous	182 311 (36.1%)	727 (22.4%)	0.31	36 921 (21.3%)	518 (21.3%)	0.00
Multiparous	105 010 (20.8%)	654 (20.1%)	0.02	29 737 (17.2%)	415 (17.1%)	0.00
Missing	3166 (0.6%)	11 (0.3%)	0.04	412 (0.2%)	8 (0.3%)	0.02
Antenatal care provider						
No	2939 (0.6%)	100 (3.0%)	0.19	1853 (1.1%)	26 (1.1%)	0.00
Yes	484 800 (96.0%)	3042 (93.7%)	0.11	168 351 (97.1%)	2362 (97.1%)	0.00
Missing	17 038 (3.4%)	106 (3.3%)	0.01	3065 (1.8%)	43 (1.8%)	0.00
Maternal smoking						
No	421 957 (83.6%)	594 (18.3%)	1.73	132 072 (76.2%)	1853 (76.2%)	0.00
Yes	58 921 (11.7%)	2539 (78.2%)	1.8	4918 (2.8%)	69 (2.8%)	0.00
Missing	23 899 (4.7%)	115 (3.5%)	0.06	36 279 (20.9%)	509 (20.9%)	0.00
Obstetric complications‡						
Placental abruption	2703 (0.5%)	44 (1.4%)	0.08	1366 (0.8%)	34 (1.4%)	0.06
Placenta previa	3231 (0.6%)	16 (0.5%)	0.02	676 (0.4%)	11 (0.5%)	0.01
Pre-eclampsia	9633 (1.9%)	62 (1.9%)	<0.01	3209 (1.9%)	49 (2.0%)	0.01
Eclampsia	177 (0.0%)	<6†	0.01	112 (0.1%)	<6†	0.01
Gestational diabetes	23 903 (4.7%)	83 (2.6%)	0.12	4702 (2.7%)	59 (2.4%)	0.02
Gestational hypertension	17 479 (3.5%)	104 (3.2%)	0.01	6458 (3.7%)	71 (2.9%)	0.05
Gestational age at delivery‡						
Preterm birth (<37 week)	31 066 (6.2%)	419 (12.9%)	0.23	13 819 (8.0%)	283 (11.6%)	0.12
Full-term birth (≥37 weeks)	473 711 (93.8%)	2829 (87.1%)	0.23	159 450 (92.0%)	2148 (88.4%)	0.12

∗ Measured using census data as the neighborhood proportion of people with a postsecondary education degree. Percentages were rounded to the nearest decimal place. Area-level median family income quintiles were extracted from the Canadian Census using patient postal codes mapped to standard geographical units for census tracts and dissemination areas. Gestational age was estimated by first-trimester ultrasound or menstrual dating.

‡ Variable not used in CEM procedure.

† Frequencies and percentages for cell counts less than 6 are suppressed.

Poisson regression was used to compute rate ratios and 95% CIs to assess the differences in the rates of long-term health service use between children with and without prenatal cannabis exposure. We first fit unadjusted models on the full sample, followed by adjusted analyses using the matched sample, and finally, fully-adjusted models within the matched sample. Using the matched sample, fully adjusted models further accounted for obstetric complications, including placental abruption, placenta previa, pre-eclampsia, eclampsia, gestational diabetes, gestational hypertension, and preterm birth. Subgroup analyses were performed using a priori-specified factors associated with prenatal cannabis use, which may also be intermediate variables affecting health service use.^4^^,^^18^ These were preterm birth (born <37 or ≥37 weeks gestation), area-level median household income (high vs low income) and substance use (any substance use vs no use).

## Results

The initial study sample comprised 689 071 pregnancy records. In total, 181 046 records were excluded due to missing information, linkage errors, being a non-Ontario resident at birth, stillbirth, multifetal pregnancies, OHIP ineligibility, maternal age above 50 years at delivery or missing information on cannabis, yielding a final analytic sample of 508 025 (Figure). Children were followed up for a median of 7 years. Total person-years were 3 713 219.88 and 22 606.70 among the unexposed and exposed groups, respectively (Supplemental Table S3; available at www.jpeds.com).

**Figure:**
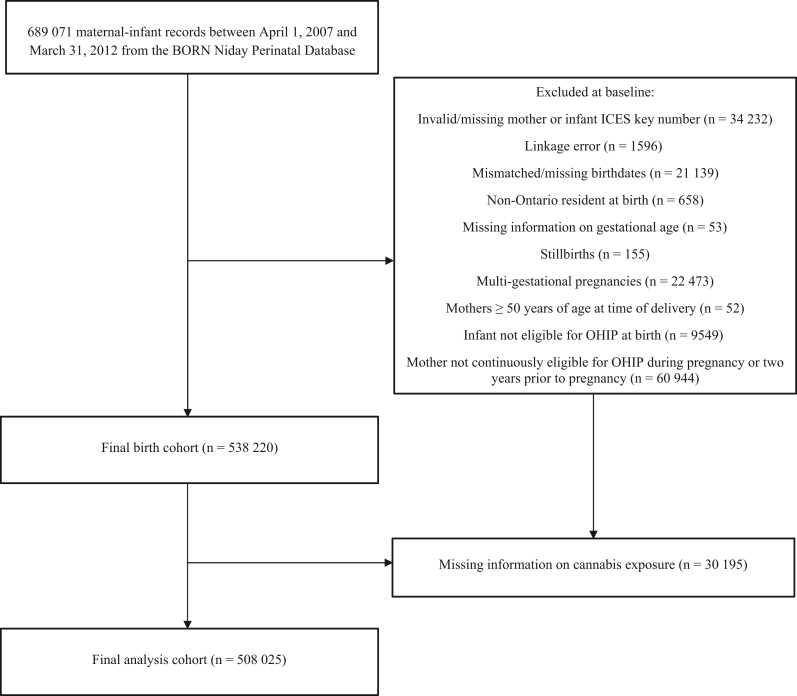
Flow diagram of population included in the analysis.

Overall, 3248/508 025 (0.6%) children were exposed to cannabis in utero, and the prevalence was highest among mothers younger than 20 (4.4% = 843/19 042). Before matching, mothers who used cannabis during pregnancy were younger (mean age 23 years vs 30 years; SMD: 1.11), less educated (SMD: 0.63) and of lower income (44.8% vs 21.0%; SMD: 0.52) compared to nonusers (Table I). After applying CEM and removing unmatched observations, our cohort included 175 700 infants (2431 with cannabis use and 173 269 without). All measured baseline covariates used in matching were well-balanced (SMD < 0.1) across exposure groups (Table I). After matching, preterm birth remained more prevalent among mothers with cannabis use compared to nonusers (11.6% vs 8.0%, respectively).

### Association Between Prenatal Cannabis Exposure and Health Service Use

After adjusting for obstetrical complications in the matched cohort, we found that offspring exposed to cannabis during the gestational period had decreased rates of primary care visits compared to those unexposed (additionally adjusted RR [a_2_RR]: 0.86, 95% CI: 0.84-0.87). However, they had increased rates of visiting an outpatient psychiatrist (a_2_RR: 1.29, 95% CI: 1.00-1.66), the ED (a_2_RR: 1.05, 95% CI: 1.03-1.08) and being hospitalized (a_2_RR: 1.12, 95% CI: 1.04-1.20) (Table II). The mean frequency of outpatient psychiatrist visits across the cohort was low among both unexposed (mean = 0.07, SMD: 1.07) and exposed (mean = 0.14, SMD: 1.09) infants (Supplemental Table S3; available at www.jpeds.com). Similarly, hospitalizations were infrequent among the cohort's unexposed (mean = 0.29, SMD: 0.97) and exposed (mean = 0.37, SMD: 1.08) children. In contrast, offspring in the cohort frequently utilized primary care and ED services throughout follow-up.

**Table II:** Association Between Prenatal Cannabis Exposure and Rate of Childhood Health Services Use by Service

Outcomes	Crude RR (95% CI) (n = 508 025)	Adjusted RR [a_1_RR] (95% CI)∗ (n = 175 700)	Additionally adjusted RR [a_2_RR] (95% CI)∗† (n = 175 700)
Primary care visits (family physicians and pediatricians)	0.79 (0.77-0.81)	0.85 (0.84-0.87)	0.86 (0.84-0.87)
Outpatient psychiatrist visits	1.94 (1.40-2.69)	1.30 (1.01-1.67)	1.29 (1.00-1.66)
Emergency department visits	1.46 (1.40-1.51)	1.06 (1.04-1.09)	1.05 (1.03-1.08)
In-patient hospitalizations	1.41 (1.30-1.54)	1.19 (1.11-1.28)	1.12 (1.04-1.20)

*RR*, rate ratio; *95% CI*, 95% confidence interval.

∗ Models use CEM sample.

† Adjusted for placental abruption, placenta previa, pre-eclampsia, eclampsia, gestational diabetes, and preterm birth.

### Stratified Analysis by Preterm Birth, Other Substance Use, and Area-Level Income

Among children born preterm (n = 31 485), the unadjusted analysis showed a similar pattern across health service outcomes compared to the overall population. However, after matching and adjustment for obstetrical complications, those exposed to prenatal cannabis had lower rates of primary care visits (a_2_RR: 0.82, 95% CI: 0.78-0.87) compared to no cannabis, while the rates of other service use were not statistically different (Table III). Among full-term infants, the cannabis-outcome associations closely aligned with the overall sample. Further, among offspring of women without additional substance use (eg, no alcohol, tobacco, cocaine, hallucinogens, methadone, or opioids), we found the rates of health service use associated with prenatal cannabis exposure remained similar to that of the overall cohort for all outcomes.

**Table III:** Association Between Prenatal Cannabis Exposure and Childhood Health Services Outcomes, by Preterm Birth, Substance Use and Income

Cohort characteristic	Outcomes	Crude RR (95% CI) (n = 508 025)	Adjusted RR [a_1_RR] (95% CI)∗ (n = 175 700)	Additionally adjusted RR [a_2_RR] (95% CI)∗† (n = 175 700)
Preterm birth				
	Primary care visits (family physicians and pediatricians)	0.73 (0.68-0.79)	0.82 (0.78-0.87)	0.82 (0.78-0.87)
	Outpatient psychiatrist visits	1.24 (0.54-2.82)	0.96 (0.46-2.01)	0.96 (0.46-2.02)
	Emergency department visits	1.24 (1.12-1.38)	0.98 (0.91-1.05)	0.98 (0.91-1.05)
	In-patient hospitalizations	1.15 (0.96-1.38)	0.96 (0.81-1.12)	0.94 (0.80-1.11)
Full-term birth				
	Primary care visits (family physicians and pediatricians)	0.79 (0.77-0.81)	0.86 (0.85-0.87)	0.86 (0.85-0.87)
	Outpatient psychiatrist visits	2.02 (1.42-2.88)	1.34 (1.02-1.75)	1.33 (1.01-1.74)
	Emergency department visits	1.47 (1.41-1.53)	1.06 (1.04-1.09)	1.06 (1.04-1.09)
	In-patient hospitalizations	1.35 (1.22-1.48)	1.16 (1.07-1.26)	1.16 (1.07-1.26)
No use of other substances‡				
	Primary care visits (family physicians and pediatricians)	0.78 (0.76-0.81)	0.84 (0.83-0.86)	0.84 (0.83-0.86)
	Outpatient psychiatrist visits	1.98 (1.34-2.93)	1.32 (1.01-1.72)	1.31 (1.00-1.71)
	Emergency department visits	1.43 (1.37-1.50)	1.04 (1.02-1.07)	1.04 (1.01-1.06)
	In-patient hospitalizations	1.34 (1.21-1.49)	1.16 (1.08-1.26)	1.09 (1.01-1.18)
Income				
Quintile: 1,2 (low income) Quintile: 3,4, 5 (higher income)	Primary care visits (family physicians and pediatricians)	0.78 (0.76-0.81)	0.87 (0.85-0.89)	0.87 (0.85-0.89)
	Outpatient psychiatrist visits	1.53 (1.04-2.24)	1.00 (0.68-1.45)	0.99 (0.68-1.44)
	Emergency department visits	1.30 (1.25-1.37)	1.01 (0.98-1.04)	1.00 (0.97-1.04)
	In-patient hospitalizations	1.31 (1.18-1.46)	1.12 (1.03-1.23)	1.06 (0.97-1.16)
	Primary care visits (family physicians and pediatricians)	0.83 (0.79-0.86)	0.85 (0.83-0.87)	0.85 (0.83-0.87)
	Outpatient psychiatrist visits	2.76 (1.51-5.01)	2.46 (1.81-3.35)	2.49 (1.83-3.38)
	Emergency department visits	1.59 (1.49-1.70)	1.17 (1.13-1.21)	1.16 (1.12-1.21)
	In-patient hospitalizations	1.37 (1.18-1.60)	1.29 (1.13-1.46)	1.21 (1.06-1.37)

*RR*, rate ratio; *95% CI*, 95% confidence interval.

∗ Models adjusted using the CEM method.

† Adjusted for placental abruption, placenta previa, preeclampsia, gestational diabetes, gestational hypertension, and preterm birth.

‡ Individuals self-reported consuming cannabis and no alcohol, cocaine, hallucinogens, methadone, or opioids.

Finally, when stratifying by lower and higher income levels, both strata had similar rates of primary care visits. The rates of outpatient psychiatrist visits among those prenatally exposed to cannabis and whose families are in the upper-income quintiles were over two times higher (a_2_RR: 2.49, 95% CI: 1.83-3.38) than those of the same income quintiles who were unexposed. In contrast, the rate of outpatient psychiatrist visits among those of lower quintiles did not differ between those exposed or unexposed (a_2_RR: 0.99, 95% CI: 0.68-1.44). Similarly, prenatal cannabis use was associated with an increased rate of ED visits (a_2_RR: 1.16, 95% CI: 1.12-1.21) and hospitalizations (a_2_RR: 1.21, 95% CI: 1.06 -1.37), only in the higher income strata.

## Discussion

In this study, prenatal cannabis use in Ontario was associated with increased rates of ED visits, hospitalizations and psychiatrist visits for offspring within the first ten years since birth. In contrast, the rate of primary care access was roughly 15% lower among these children compared to children with no prenatal cannabis exposure. Prenatal cannabis use was associated with lower rates of primary care visits regardless of income level or prematurity. However, associations with other health services differed by income level and prematurity.

In line with this, Avalos et al^31^ found that in-utero exposure to cannabis was associated with a significantly increased risk of missed well-child visits in the first 3 years of life of the offspring, in the United States. Decreased rates of primary care visits in childhood may indicate that, where maternal cannabis use is identified, children and their families face increased barriers to accessing pediatricians or primary care providers due to factors such as fear of stigmatization,^9^^,^^32^ reduced access to physicians,^33^ inability to take time off work or mental health difficulties.^34^ Not having a primary care physician or having difficulty accessing primary care has been found to increase the likelihood of visiting an emergency room and hospital admissions,^35^^,^^36^ which was observed in this cohort. Furthermore, unfavorable psychosocial and socioeconomic determinants of health are more prevalent among mothers who use cannabis during pregnancy.^4^^,^^26^ As a result, these mothers may experience disparities in health services use and quality,^37^, ^38^, ^39^ and this may persist as they seek appropriate care for their children after birth.

Previous studies have found that prenatal exposure to cannabis through maternal use can cause decreases in cognitive function among offspring.^40^, ^41^, ^42^ Using a similar cohort, we found that the risk of autism was 50% higher for children exposed to cannabis in utero compared to those unexposed.^26^ A study from Australia reported a 3-fold increase in risk of autism diagnosis among offspring of mothers diagnosed with cannabis use disorder.^43^ Evidence is still emerging, and several cohort studies have been null. However, a 2024 meta-analysis of 4 studies and ∼200 000 participants found a pooled rate ratio of 1.3 (95% CI: 1.03-1.64) for the association between in-utero cannabis exposure and autism in offspring. Psychological perturbations caused by exogenous cannabinoids during development may explain the higher rates of psychiatric visits seen among those in this cohort who were prenatally exposed to cannabis. Consistent with our findings, prenatal cannabis exposure is typically more common among infants born to lower income families.^4^^,^^44^ Interestingly, outpatient psychiatrist visit rates did not differ by cannabis use among individuals of the lower income quintiles. In contrast, rates were much higher among higher income children with prenatal exposure. This difference might be explained by the difference in socioeconomic position, where parents in higher income quintiles are more aware of the risk of developmental conditions and have more flexibility to take time off work or more resources to access private care and pay for private services through private insurance. Furthermore, children living in neighborhoods of higher median income may be placed in schools that are more aware and possess the resources to monitor for behavioral problems or learning disabilities, which may direct them to seek health care specialists if necessary.

### Limitations

In this study, cannabis exposure was a binary variable and did not account for variation in frequency or intensity of exposure during the prenatal period, which could differentially impact the development of exposed children. Data on the use of cannabis in pregnancy are self-reported and collected through disclosure to prenatal care providers using a standardized form during routine perinatal care. Cannabis use data in the BORN perinatal database are highly accurate when validated against clinical records, although similar biases may exist in both sources.^18^ Our cohort was assembled in an early period when the prevalence of cannabis use was lower and the methods for cannabis data collection were less well-established. Misclassification bias remains due to under-reporting of cannabis use in pregnancy, which may arise from social stigma or fear of involvement of child protective services.^45^, ^46^, ^47^ However, given that the exposure may be under-reported in this cohort, the associations we describe here may underestimate rather than exaggerate the relationship between cannabis use in pregnancy and child health service use. Our cohort also predates the legalization of nonmedical cannabis in Canada (October 2018), although the data are highly relevant for understanding prelegalization patterns of exposure and health service use. Further, data were collected before some increases in potency and product availability. However, the delta-9 tetrahydrocannabinol potency in cannabis products during this time was already beginning to increase compared to prior decades.^48^ Ongoing surveillance and replication using postlegalization data are warranted to assess how changing regulatory and social contexts may influence these associations. Children were followed for a maximum of 10 years, with a median follow-up of 7 years. Since prenatal cannabis use often results in health conditions that require more medical attention immediately after birth rather than later in childhood, health service use patterns may differ by different timing in the life course. Previous studies have found that children exposed to cannabis during gestation are at a higher risk of neonatal intensive care unit admission and transfer.^17^^,^^18^^,^^20^^,^^24^ The number of hospitalizations, for example, is likely disproportionately higher within the first months since birth. Given the long-term follow-up, there is also a possibility of unmeasured confounders that may occur later in life. Future research should focus on assessing service use in different age intervals for a deeper understanding of the pattern of service use throughout childhood.

### Interpretation and Generalizability

This study is among the first in Canada to evaluate the relationship between prenatal cannabis exposure and the frequency of health care visits in childhood up to 10 years of age. These findings underscore that prenatal cannabis use may be associated with ongoing changes to health care use patterns in offspring past infancy. Clinical practice should continue to reinforce the importance of safe and respectful dialog between expectant mothers and health care professionals regarding cannabis use during pregnancy. Early screening and identification of prenatal cannabis use, along with appropriate follow-up, may ensure timely assessment of affected children who may be more vulnerable to adverse social and health outcomes. We recommend developing publicly accessible guidelines for the prenatal consumption of cannabis, such as those recently released by the American Society of Clinical Oncology for cancer patients and physicians,^49^ which provides strategies for open and nonjudgmental communication between clinicians and patients around cannabis use. Such guidelines may be used to help reduce potentially harmful cannabis consumption and potentially contribute to reducing the long-term impacts of prenatal cannabis use on health service use.

## Data Statement

The dataset used in this research is held securely in coded form at ICES. Requests for data access can be made to ICES at https://www.ices.on.ca/services-for-researchers/submit-a-request/. The complete dataset creation plan is available from the authors upon request.

## Disclaimers

This study is based on data from the Better Outcomes Registry & Network (“BORN”), part of the Children's Hospital of Eastern Ontario. Parts of this material are based on data and information compiled and provided by the Canadian Institute for Health Information and the Ontario Ministry of Health. The analyses, conclusions, opinions, and statements expressed herein are solely those of the authors and do not reflect those of the funding or data sources; no endorsement is intended or should be inferred. We use data adapted from the Statistics Canada Postal Code^OM^ Conversion File, based on data licensed from Canada Post Corporation, and/or data adapted from the Ontario Ministry of Health Postal Code Conversion File, which contains data copied under license from Canada Post Corporation and Statistics Canada. This does not constitute an endorsement by Statistics Canada of this product.

## CRediT authorship contribution statement

**Gabrielle Pratt Tremblay:** Writing – original draft, Investigation. **Arum Han:** Writing – original draft, Investigation. **Ewa Sucha:** Writing – review & editing, Formal analysis, Data curation. **Helen Hsu:** Writing – review & editing, Investigation. **Jessy Donelle:** Writing – review & editing, Formal analysis, Data curation. **Daniel J. Corsi:** Writing – review & editing, Supervision, Methodology, Investigation, Funding acquisition, Conceptualization.

## Declaration of Competing Interest

The authors declare that they have no known competing financial interests or personal relationships that could have appeared to influence the work reported in this paper. This study was funded by the Canadian Institutes of Health Research.
