# Corrigendum to Association Between Prenatal Cannabis Exposure and Child Health Care Use: A Retrospective Cohort Study in Ontario, Canada

**DOI:** 10.1016/j.jpedcp.2025.200171

**Published:** 2025-07-29

**Authors:** Gabrielle Pratt Tremblay, Arum Han, Ewa Sucha, Helen Hsu, Jessy Donelle, Daniel J. Corsi

**Affiliations:** 1 School of Epidemiology and Public Health, Faculty of Medicine, University of Ottawa, Ottawa, Ontario, Canada; 2 Children's Hospital of Eastern Ontario Research Institute, Ottawa, Ontario, Canada; 3 Faculty of Medicine, University of Ottawa, Ottawa, Ontario, Canada; 4 ICES uOttawa, Ottawa, Ontario, Canada; 5 Kingston Health Sciences Centre, Kingston, Ontario, Canada

The authors regret that the following acknowledgement was omitted from the originally published article:

## ICES Funding Acknowledgement

This study was supported by 10.13039/100012665ICES, which is funded by an annual grant from the Ontario 10.13039/100009647Ministry of Health (MOH) and the Ministry of Long-Term Care (MLTC).

