# Supplementary material for: Association Between Prenatal Cannabis Exposure and Child Health Care Use: A Retrospective Cohort Study in Ontario, Canada
Source: J Pediatr Clin Pract. 2025 Jun 2;17:200151. doi: 10.1016/j.jpedcp.2025.200151 (PMC12206123; doi:10.1016/j.jpedcp.2025.200151)
Supplement: Supplementary Materials [file mmc1.docx]

**SUPPLEMENTAL APPENDIX**

Pratt Tremblay G, Han A, Sucha E, Hsu H, Donelle J, Corsi DJ, "Association Between Prenatal Cannabis Exposure and Child Health Care Use: A Retrospective Cohort Study in Ontario, Canada"

**Methods**

**Data sources**

The following databases were used to ascertain covariates and outcomes: 1) the Registered Persons Database (RPDB), which contains demographic data and coverage periods for all individuals registered in the provincial insurance plan. ICES maintains a master registration database for all Ontario residents with a valid provincial health card. These files contain demographic data for anyone who has received an Ontario health card number and indicate the eligibility period for individuals to receive provincially-funded health care, accounting for deaths or migration out of province; 2) the Ontario Health Insurance Plan (OHIP) database, which stores physician billing information from primary care or outpatient physician visits across the province; 3) the Canadian Institute for Health Information (CIHI) Discharge Abstract Database (DAD) for Ontario, which captures information on hospitalizations, length of stay, with associated ICD-codes for diseases, and health conditions; and 4) the National Ambulatory Care Reporting System (NACRS) for Ontario, managed by CIHI, which captures data on hospital visits (including hospital-based clinics or emergency department visits) or community-based ambulatory care that does not require overnight admission.^1,2^

**References**

1. Wilson K, Hawken S, Kwong JC, Deeks SL, Crowcroft NS, Manuel D. Vaccine and Immunization Surveillance in Ontario (VISION) - using linked health administrative databases to monitor vaccine safety. *Vaccine.* 2012;30:6115-20.

2. Schwartz KL, Tu K, Wing L, et al. Validation of infant immunization billing codes in administrative data. *Hum Vaccin Immunother.* 2015;11:1840-7.

**Supplemental Table S1.** Cohort inclusion and exclusion criteria

| **Characteristics** | **Inclusion** | **Exclusion** |
| --- | --- | --- |
| Participants: Age | 16-50 years of age | <16 years of age  >50 years of age |
| Participants: residence | Infants born to Ontario residents | Infants born to non-Ontario residents  Infants born to individuals who did not reside in Ontario for at least 2 years prior to pregnancy |
| Participants: infants | Singleton birth between April 1, 2007-March 31, 2012 | Multi-fetal birth  Birth before April 1, 2007 or after March 31, 2012  Infants with implausible birthweight or gestational age  Infants with invalid date of death  Infants with no follow-up time |
| Administrative | Infants eligible for OHIP at birth  Mothers with continuous OHIP eligibility from 3 months before conception until date of birth | Infants ineligible for OHIP at birth  Infants or mothers with data linkage errors  Mothers without continuous OHIP eligibility from 3 months before conception until date of birth |
| Exposure | Prenatal cannabis use (yes/no) | Missing information on cannabis use |
| Outcomes | Primary care physician visit  Outpatient psychiatrist visit  Emergency department visit  Hospitalization | n/a |

**Supplemental Table S2** Primary care “core” OHIP fee codes

The following OHIP fee codes were used to identify primary care outpatient visits (including additional pediatric codes):

| **Code** | **Description** |
| --- | --- |
| A001 | Minor assessment |
| A002 | 18-month well-baby check |
| A003 | General assessment |
| A007 | Intermediate assessment or well-baby care |
| A261 | Pediatric assessment level 1 (minor assessment) |
| A268 | Enhanced 18-month well-baby visit |
| A903 | Pre-dental/pre-operative general assessment |
| G212 | Allergy injection alone |
| G271 | Anticoagulant supervision |
| G365 | Papanicolaou test |
| G372 | Injection, with visit |
| G373 | Injection, sole reason |
| G538 | Immunization, with visit |
| G539 | Immunization, sole reason |
| G590 | Influenza immunization, with visit |
| G591 | Influenza immunization, sole reason |
| K005 | Primary mental healthcare |
| K013 | Counselling, individual care |
| K017 | Annual health exam, child after second birthday |
| K130 | Adolescent periodic health visit |
| K131 | Adult periodic health visit age 18–64 |
| K132 | Adult periodic health visit over age 65 |
| K267 | Annual health exam, child 2–11 years, after 2nd birthday |
| K269 | Annual adolescent health exam |

**Supplemental Table S3.** Frequency and total person-years of primary and secondary health service visits among unexposed and exposed infants in the entire birth cohort and CEM cohort.

| **Outcomes** | **Birth cohort by prenatal cannabis exposure**  **(N = 508,025)** | | | | | | **CEM (Matched) cohort by prenatal cannabis exposure**  **(N = 175,700)** | |
| --- | --- | --- | --- | --- | --- | --- | --- | --- |
|  | **Unexposed**  **(n = 504,777)** | | | **Exposed**  **(n = 3,248)** | | | **Unexposed**  **(n = 173,269)** | **Exposed**  **(n = 2,431)** |
|  | **No. events** | **Mean (SD)** | **Total person-years** | **No. events** | **Mean (SD)** | **Total person-years** | **Mean**  **(SD)** | **Mean**  **(SD)** |
| Primary care visits (family physicians and pediatricians) | 11,163,357 | 22.12 (15.80) | 3,713,219.88 | 52,346 | 16.12 (12.41) | 22,606.70 | 22.46 (15.74) | 16.47 (12.16) |
| Outpatient psychiatrist visits | 36,630 | 0.07 (1.07) | 3,713,219.88 | 444 | 0.14 (1.09) | 22,606.70 | 0.07 (1.28) | 0.14 (1.14) |
| Emergency department visits | 2,051,786 | 4.06 (4.98) | 3,713,219.88 | 17,908 | 5.51 (5.98) | 22,606.70 | 4.34 (5.11) | 5.45 (5.95) |
| In-patient hospitalizations | 145,329 | 0.29 (0.97) | 3,713,219.88 | 1,203 | 0.37 (1.08) | 22,606.70 | 0.29 (0.99) | 0.35 (1.08) |
